# Editorial: Training load in sport: current challenges and future perspectives

**DOI:** 10.3389/fspor.2025.1574500

**Published:** 2025-02-21

**Authors:** Luís Branquinho, Pedro Forte, Elias de França, Ricardo Ferraz, José Eduardo Teixeira, Ronaldo Thomatieli-Santos

**Affiliations:** ^1^ Biosciences Higher School of Elvas, Polytechnic Institute of Portalegre, Portalegre, Portugal; ^2^ Life Quality Research Center (LQRC-CIEQV), Santarém, Portugal; ^3^ Research Center in Sports, Health and Human Development, Covilhã, Portugal; ^4^ CI-ISCE, Instituto Superior de Ciências Educativas do Douro (ISCE Douro), Penafiel, Portugal; ^5^ Sports Department, Higher Institute of Educational Sciences of the Douro, Penafiel, Portugal; ^6^ LiveWell—Research Centre for Active Living and Wellbeing, Polytechnic Institute of Bragança, Bragança, Portugal; ^7^ Department of Sports Sciences, Polytechnic Institute of Bragança, Bragança, Portugal; ^8^ Interdisciplinar Graduate Program in Health Sciences, Universidade Federal de São Paulo, Santos, Brazil; ^9^ Human Movement Laboratory, São Judas University, São Paulo, Brazil; ^10^ Sport Sciences Department, University of Beira Interior, Covilhã, Portugal; ^11^ Department of Sports, Polytechnic of Guarda, Guarda, Portugal; ^12^ Department of Sports Sciences, Polytechnic of Cávado and Ave, Guimarães, Portugal; ^13^ SPRINT—Sport Physical Activity and Health Research & Inovation Center, Guarda, Portugal; ^14^ Graduate Program in Psychobiology, Universidade Federal de São Paulo, São Paulo, Brazil

**Keywords:** training load, athletic performance, injury prevention, strength and power development, recovery strategies

**Editorial on the Research Topic**
Training load in sport: current challenges and future perspectives

## Theoretical framework

Training load is a critical component of athletic development, serving as a fundamental determinant of performance enhancement and injury prevention (1). Factors such as training intensity, volume, frequency, and density must be carefully managed to promote positive adaptations in athletes (2). The concept of training load is not merely a measure of the amount of work performed, it is a complex interplay of factors that can significantly influence an athlete's performance trajectory (3). Understanding how to optimize training load is essential to maximizing athletic performance while minimizing the risks of excessive fatigue, injury, and overtraining, which can negatively impact an athlete's performance and ability to compete and train effectively, as well as overall health (1).

Recent research has demonstrated a clear relationship between increasing training loads and the incidence of injuries, particularly in high-impact sports where the risk of cumulative trauma is increased (4, 5). Therefore, a comprehensive understanding of training load dynamics is crucial for coaches and athletes to enable a balance to be found between performance thresholds and injury risk.

Recent advances in technology and data analytics have revolutionized the way training loads are monitored and managed. The integration of wearable devices and software applications allows real-time tracking of an athlete's physiological responses to training, providing valuable insights into their recovery needs and overall readiness to train (6). This type of data-driven approach facilitates the creation of individualized training programs that consider physical, physiological, and psychological profiles, and that consequently promote training satisfaction and reduce the risk of (7). Furthermore, the emphasis on individualized training loads is aligned with contemporary training philosophies that advocate athlete-centered training methodologies, where the athlete's contribution and experiences are essential for the optimization of the training process (8).

To clarify and further explore these issues, this Research Topic, Training Load in Sport: Current Challenges and Future Perspectives, presents a collection of studies that explored the current perspective on knowledge and challenges associated with the effects of careful manipulation and management of load to optimize performance and promote health in athletes across different sports and competitive levels.

## Current challenges and future perspectives

Throughout this research topic, there were numerous contributions to investigate the current state and future perspectives in relation to training load in sport. Tilp et al. investigated the relationship between systemic and local muscle breaking points in single-leg cycling, finding strong correlations but significant individual variability. Similarly, Kårström et al. revealed discrepancies between internal and external load assessments in biathlon, suggesting that a multimodal approach is necessary for accurate monitoring. Masur et al. explored infrared thermography as a non-invasive tool to track internal burden, although inconsistencies in its relationship with traditional markers indicate that further validation is needed. Meanwhile, studies on training methods, such as those by Wei and Zheng and Quan et al., showed that small-sided games (SS) and high-intensity interval training (HITT) can generate varied benefits, especially for athletes with lower physical conditioning. Sheykhlouvand and Gharaatin in turn, analysed adaptations in cardiorespiratory fitness and biomotor skills in soccer players trained with short sprint interval training (sSIT) SSG. The sSIT promoted more homogeneous responses in ventilatory thresholds, stroke volume, and maximal power, while the SSG showed lower proportions of responders in maximal oxygen uptake, ventilatory thresholds, and anaerobic power, suggesting greater effectiveness of sSIT for consistent adaptations. Furthermore, Talsnes et al. found that splitting moderate-intensity training into two shorter sessions reduces physiological stress while maintaining training adaptations.

Physiological responses to training load go beyond performance outcomes, influencing vascular function, muscular adaptations and recovery strategies. Sugawara et al. observed that football matches induced transient reductions in arterial wave reflection without increasing arterial stiffness, suggesting adaptive responses to repeated exposure to matches. Similarly, Yu et al. recommended periodized HIIT, sprint, and threshold training for sedentary youth to maximize cardiovascular benefits while avoiding overload. Studies on strength and power development have also provided insights into how to optimize training stimuli. Cui et al. identified specific velocity loss thresholds that enhance post-activation potentiation effects in boxers. Naczk et al. demonstrated that inertial training offers small advantages over traditional resistance training for knee extensor strength. Meanwhile, Singer et al. pointed out that rest intervals longer than 60 s may provide additional hypertrophic benefits, especially beyond 90 s. Ma et al. found that blood flow restriction training may be a viable alternative to conventional strength training, offering similar improvements in muscle strength and thickness.

Injury prevention and recovery strategies are essential components of effective training load management. Huang et al. examined whole-body cryotherapy (WBC) in elite rowers, concluding that although WBC accelerates blood lactate clearance, it does not significantly improve overall recovery. Xie et al. further demonstrated that HIIT is more effective than moderate-intensity continuous training in improving post-exercise lactate clearance. In the context of injury prevention, Iwasaki et al. established a strong link between contact load and injury risk in elite rugby players, emphasizing the importance of monitoring acute and chronic workload ratios. Reverte-Pagola et al. analysed LaLiga soccer players who did not participate in the FIFA World Cup, finding that optimized load management during the tournament break led to improved sprint and acceleration performance. Furthermore, Cui et al. demonstrated that load-adjusted strength training improves punching capacity and energy efficiency in elite female boxers more effectively than traditional methods.

Finally, methodological considerations in training load research require further refinement to ensure robust conclusions. de Queiros et al. critically evaluated the systematic review by Ma et al. on BFR training, highlighting concerns related to study selection, assessment of risk of bias, and heterogeneity in comparative studies. These methodological challenges highlight the need for standardized approaches in training load research, ensuring that practitioners and researchers can develop evidence-based strategies tailored to individual athlete needs.

Studies in this Research Topic provide critical and innovative insights into training load monitoring, adaptation, and injury prevention. Advances in non-invasive monitoring tools, training periodization, and recovery strategies continue to shape evidence-based practices for optimizing athlete performance. Future research should explore individualized training load prescriptions, integrating physiological, biomechanical and technological innovations. Professionals in the field can refine training programs to achieve the best results for athletes by incorporating multifaceted monitoring strategies.
